# Dose-Registered CT assessment of radiation-induced lung injury after chemoradiotherapy for stage III NSCLC

**DOI:** 10.1080/07853890.2026.2721850

**Published:** 2026-09-10

**Authors:** Tomohiro Itonaga, Ryuji Mikami, Tatsuhiko Zama, Mitsuru Okubo, Shinji Sugahara, Norihiko Ikeda, Kazuhiro Saito

**Affiliations:** a Department of Radiology, Tokyo Medical University, Shinjuku, Tokyo, Japan; b Department of Radiology, Tokyo Medical University Hachioji Medical Center, Hachioji, Tokyo, Japan; c Department of Radiology, Tokyo Medical University Ibaraki Medical Center, Ibaraki, Japan; d Division of Thoracic and Thyroid Surgery, Tokyo Medical University, Shinjuku, Tokyo, Japan

**Keywords:** Radiation-induced lung injury, radiation pneumonitis, non-small cell lung cancer, chemoradiotherapy, deformable image registration, computed tomography, dose-volume histogram, durvalumab

## Abstract

**Aims:**

To quantify radiation-induced lung injury (RILI) on deformably registered CT images after chemoradiotherapy (CCRT) for stage III NSCLC, and to evaluate spatial and dosimetric associations between RILI and planned dose distribution in a pre-durvalumab cohort.

**Methods:**

This retrospective study included 58 patients with stage III NSCLC treated with CCRT using three-dimensional conformal radiotherapy (3D-CRT) prior to the era of consolidation immunotherapy. Follow-up CT images at 6 months (acute) and 12 months (late) were registered to treatment planning CT using deformable image registration (DIR). RILI was quantified by segmented volume (mL) and isodose-line overlap (%). Dosimetric parameters (V5Gy–V60Gy, mean lung dose) and symptomatic RILI (CTCAE grade ≥2) were analysed using correlation, ROC, and logistic regression methods.

**Results:**

Symptomatic RILI occurred in 14 patients (24.1%). RILI volume decreased by 27.7% from the acute to the late phase, with moderate-to-strong dose–volume correlations in both phases (Spearman ρ = 0.69–0.76). Symptomatic patients showed broader spatial spread into lower-dose regions (median isodose-line overlap 20% vs 40%; *p* < 0.001). Acute-phase RILI reaching the ≤25% isodose line (≤16.5 Gy) was an exploratory imaging marker in this cohort (AUC 0.81) that remained associated with symptomatic RILI on multivariable analysis (*p* < 0.01).

**Conclusions:**

Dose-registered CT analysis demonstrated consistent spatial associations between planned dose and RILI, providing a hypothesis-generating dosimetric-radiographic reference dataset that requires external validation in IMRT/VMAT- and durvalumab-treated cohorts before clinical application.

## HIGHLIGHTS

- Dose-registered CT was used to quantify radiation-induced lung injury (RILI) after definitive CCRT for stage III NSCLC in a uniform pre-durvalumab 3D-CRT cohort.
- RILI volume decreased by approximately 28% from the acute to the late phase, with consistent dose–volume correlations at both time points.
- Acute-phase RILI reaching the ≤25% isodose line (≤16.5 Gy for a 66-Gy prescription) was an exploratory imaging marker for symptomatic RILI in this cohort (AUC 0.81) that remained significant on multivariable analysis.
- This pre-durvalumab 3D-CRT cohort provides a dosimetric-radiographic reference for future studies evaluating combined radiation and immunotherapy-related pulmonary toxicity.

## Introduction

Radiation-induced lung injury (RILI) is one of the most common adverse events of concurrent chemoradiotherapy (CCRT) for patients with stage III non-small cell lung cancer (NSCLC). RILI is classified into the acute phase of radiation pneumonitis (RP), which develops late in the course of irradiation until approximately 6 months after the end of irradiation, and the late phase of radiation fibrosis (RF), which follows the acute phase [1]. Recent clinical trials have reported that the incidence of RP in patients undergoing radical CCRT for stage III NSCLC is approximately 25% [2].

Previous studies have identified risk factors for RP, including patient-related (age, sex, smoking history, interstitial lung disease), treatment-related (concomitant therapy, dosimetric parameters), and tumour-related factors (location, volume, extent of disease) [3–6]. Although multiple risk factors are involved in RILI incidence, the dosage prescribed to the lungs during radiotherapy planning has become increasingly prominent. The Quantitative Analyses of Normal Tissue Effects in the Clinic project reviewed more than 70 reports, and found that the incidence of severe RP after radiation therapy for lung cancer is related to mean lung dose (MLD) and the percentage of lung volume irradiated above a certain dose to total lung volume (V_dose_), with V20Gy to ≤30–35% and MLD to ≤20–23 Gy being recommended [3]. In recent years, lower-dose involvement has also received attention, and indices such as V5Gy and absolute volume spared from 5 Gy have been incorporated into planning [4].

Lung dose is a significant predictor of symptomatic RILI; however, its association with radiographic imaging changes is equivocal. Researchers at Duke University observed radiographic imaging changes in RILI, reporting a dose-dependent reduction in lung perfusion on single-photon emission computed tomography (SPECT) imaging and a dose-dependent increase in lung density on computed tomography (CT) imaging [7]. To build upon these findings and to enable precise comparison between treatment planning data and follow-up CT images, we employed deformable image registration (DIR), a technique that generates and applies deformation vectors to match each voxel between two imaging datasets [8].

In the current era, consolidation immunotherapy following CCRT has become the standard of care for unresectable stage III NSCLC, and treatment-related pneumonitis is often multifactorial. In the PACIFIC era, distinguishing radiation pneumonitis from immune-checkpoint-inhibitor-associated pneumonitis has become a major diagnostic challenge, as the two can be morphologically indistinguishable on CT. A uniform pre-immunotherapy cohort, therefore, provides a reference against which combined-modality toxicity can be interpreted. However, accurate characterisation of pure radiation-induced lung injury remains essential as a reference for interpreting post-treatment imaging findings and for disentangling overlapping pulmonary toxicities. To the best of our knowledge, no previous study has comprehensively examined the spatial and dosimetric correlates of RILI using DIR-based CT analysis in a uniform cohort of stage III NSCLC patients treated with definitive CCRT prior to the introduction of consolidation immunotherapy. In this retrospective study, we aimed to quantify RILI using dose-registered CT analysis and to investigate its spatial and dosimetric relationships in this reference cohort, accounting for key factors that influence RILI assessment including total dose, irradiation technique, and concurrent chemotherapy.

## Materials and methods

### Patient eligibility

**Figure 1.**
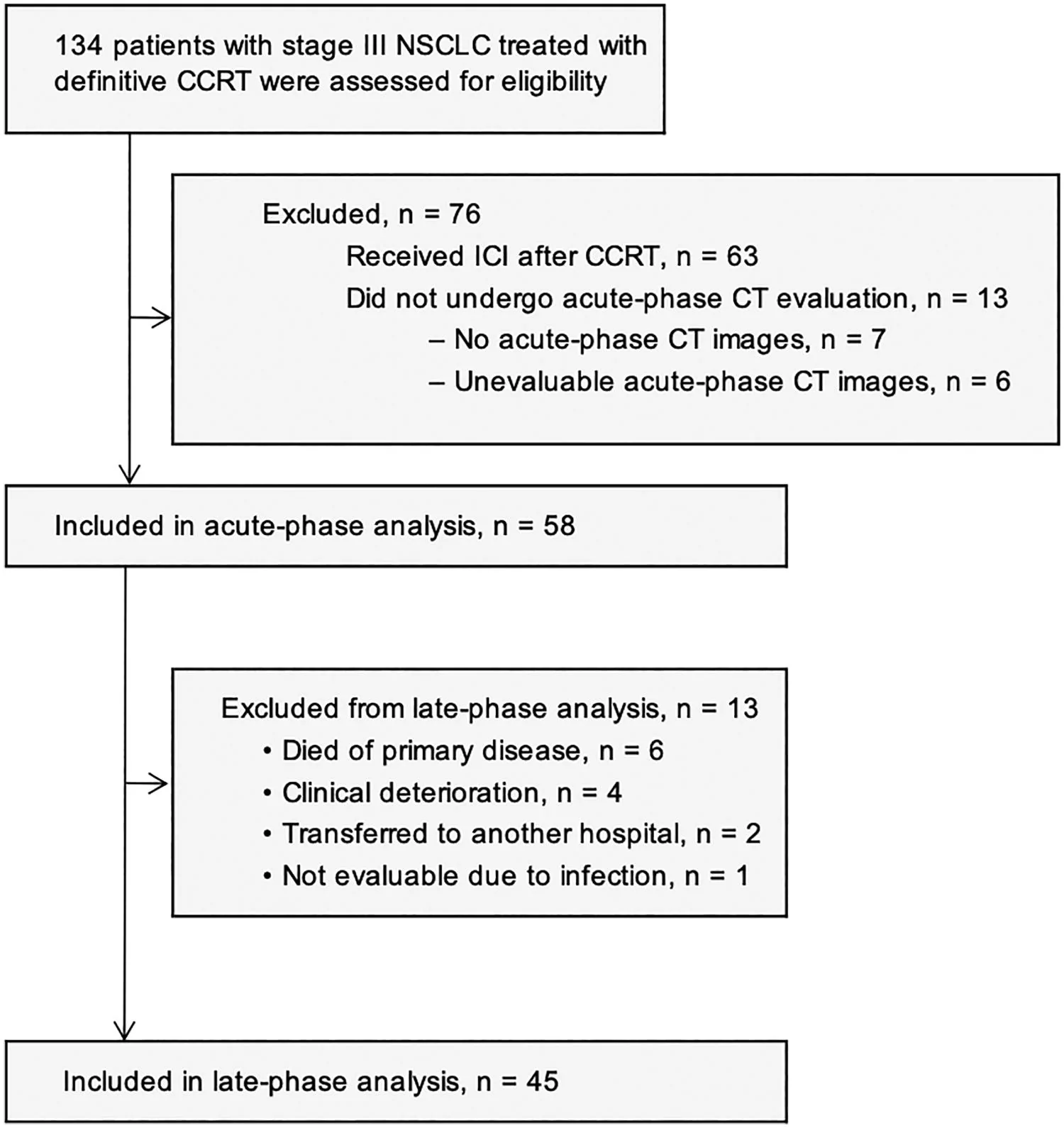
Flowchart of patient enrolment and follow-up CT availability.

This study is reported in accordance with the Strengthening the Reporting of Observational Studies in Epidemiology (STROBE) reporting guideline for observational studies. This retrospective study was approved by the Institutional Review Board of our facility (T2023-0193). Data analysis began on February 9, 2024, concurrent with the date of ethics approval. All patients had provided written informed consent permitting the use of their clinical records and imaging data for research and academic publication at our institution. Given the retrospective nature of this study, including patients who had died before study initiation, the Institutional Review Board approved an opt-out approach in place of obtaining additional study-specific written informed consent. Study information was publicly disclosed on the Tokyo Medical University Hospital website, and patients were provided the opportunity to opt out. The eligibility criteria for patients included those who underwent definitive CCRT (with a total dose of 60 Gy or more) at our institution since 2014, followed by at least 6 months of post-treatment observation. Cases in which immune checkpoint inhibitors (ICIs) were used after CCRT were excluded, because ICI-associated pneumonitis can be morphologically indistinguishable from RILI on CT imaging, and its superimposition would have confounded the dosimetric-radiographic correlations that were the primary aim of this study. Between January 2014 and January 2023, we analysed 58 patients diagnosed with stage III NSCLC according to the 8th edition of the Union for International Cancer Control ‘Tumour, Node, Metastasis’ classification. A flowchart of the enrolled patients is shown in Figure 1. Smoking history was assessed in all patients, and smoking exposure was expressed as pack-years, defined as the number of 20-cigarette packs smoked per day multiplied by the years of smoking.

### Treatment strategy

All patients in this historical cohort were treated using three-dimensional conformal radiotherapy (3D-CRT), with a daily dose of 2 Gy and a total dose of 66 Gy. The treatment planning system utilised was Xio^™^ (Elekta AB, Stockholm, Sweden) from January 2014 to June 2019 and Eclipse^™^ (Varian Medical Systems, Palo Alto, CA) from July 2019 to January 2022. As part of treatment planning, all patients underwent thoracic CT scans with a slice thickness of 2.5 mm under free-breathing conditions. CT imaging was performed with abdominal compression, essentially for the respiratory motion management of lung cancer. The target volumes were delineated as follows: gross tumour volume (GTV) was defined as the primary lesion, including enlarged regional lymph nodes identified on positron emission tomography (PET)-CT and contrast-enhanced CT images. For the clinical target volume (CTV), a margin of 5 mm was added to the GTV, excluding elective nodal irradiation. The planning target volume (PTV) was created by applying a standard 5-mm margin to the CTV. The lung volume, classified as an organ at risk, was defined as the bilateral lung parenchyma, excluding the GTV. The initial treatment plan was delivered without modification if the spinal cord maximum dose was maintained below 50 Gy and the lung V20Gy remained under 35%. If these constraints could not be achieved, re-planning was performed at 40 Gy to meet the tolerance criteria. In addition, re-planning was also carried out as appropriate when tumour-associated atelectasis either resolved or progressed. All patients received photon irradiation delivered 5 days per week, using coplanar beam arrangements of four or more fields, with 10-MV photons in the majority of cases and 6-MV photons in a minority. Re-planning was performed in 35 of 58 patients (60.3%), with five patients undergoing two re-plans. For all patients, planned dose distributions from all treatment plans were accumulated in MIM Maestro using deformable registration to form a treatment-course composite planned dose, and all dose–volume parameters (V5Gy–V60Gy and MLD) were derived from this accumulated planned dose rather than from any single plan. The severity of RILI in the acute phase was evaluated according to the Common Terminology Criteria for Adverse Events (CTCAE) version 5.0. Symptomatic RILI was graded according to CTCAE v5.0 on the basis of clinical symptoms and treatment requirements rather than imaging extent. In this cohort, all grade 2 cases required oral corticosteroids, whereas grade 3 cases required hospitalisation, supplemental oxygen, and corticosteroids. Grade ≥2 was defined as symptomatic RILI.

### Measurement method

In this study, and in accordance with the prior research, we defined RILI on CT as follows: acute RILI comprised ground-glass opacity (GGO) and/or patchy, bandlike consolidation spatially corresponding to the irradiated field and occurring within approximately 6 months from radiotherapy (RT) initiation, whereas late RILI comprised fibrotic changes – parenchymal volume loss, linear scarring/reticulation, traction bronchiectasis, and architectural distortion – assessed on CT obtained approximately 12 months after RT initiation. All CT scans were consensus-reviewed on lung-window settings by two radiation oncologists; detailed morphologic definitions and examples are provided in Supplementary Table S1 [9,10].

RILI was identified based on characteristic post-radiotherapy CT morphology, temporal evolution, and its spatial relationship to the irradiated lung. Field conformity was considered as one component of the adjudication, but competing diagnoses were excluded using an integrated clinical-radiological review, including serial CT changes, tumour growth or new lesions, bronchial obstruction or volume loss suggesting atelectasis, clinical signs of infection, laboratory findings, antibiotic response, and follow-up imaging when available; final classification was reached by consensus between two radiation oncologists, with the second reviewer blinded to symptomatic outcome and dose–volume analysis results.

**Figure 2.**
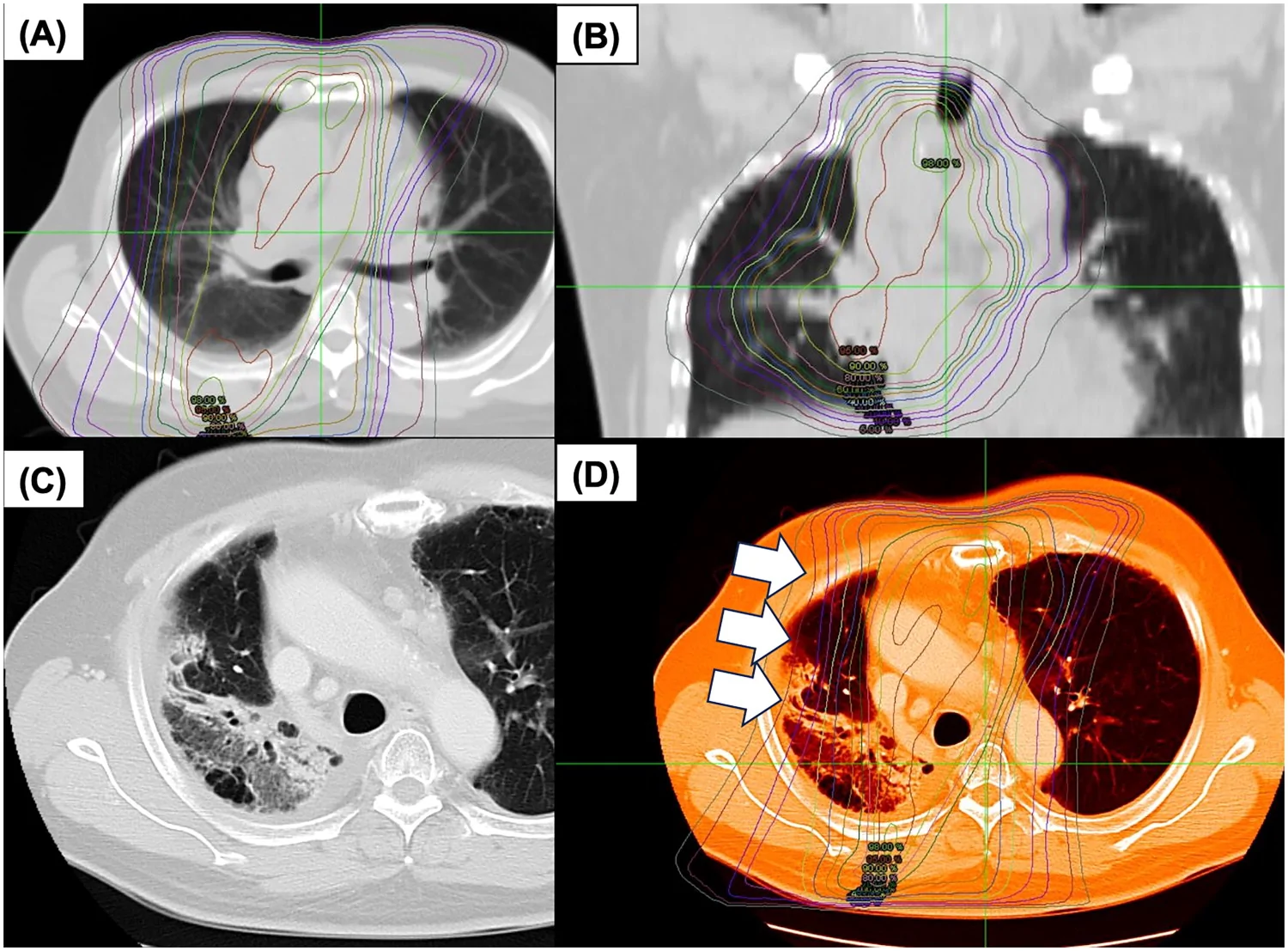
A representative case of stage III NSCLC treated with concurrent chemoradiotherapy. The background lung showed mild emphysematous changes, with a planning target volume of 335.3 mL, V_5_Gy of 33.2%, V_20_Gy of 26.1%, and mean lung dose of 15.3 Gy. Follow-up CT images were deformably registered to the treatment-planning CT and overlaid with the treatment-course composite planned dose distribution, allowing assessment of the spatial relationship between RILI and isodose lines. Registration accuracy was checked using five pulmonary-vessel landmarks and visual quality assurance of the deformed dose overlay. (A) Treatment-planning CT (axial) with dose distribution. (B) Treatment-planning CT (coronal) with dose distribution. (C) Acute-phase CT image obtained 5.7 months after treatment initiation shows confluent consolidation with traction bronchiectasis and surrounding ground-glass opacity within the irradiated field. The patient developed dyspnea and required oxygen supplementation, consistent with CTCAE grade 3. (D) Deformable image registration of (C) onto the treatment plan demonstrates that the affected RILI extends into low-dose regions, reaching approximately the 10% isodose line (white arrows).

To evaluate the isodose-line overlap and volume of RILI after CCRT, follow-up CT images obtained at approximately 6 months (acute phase) and 12 months (late phase) after the initiation of radiotherapy were overlaid onto the treatment plan using DIR technology for assessment (Figure 2). The DIR procedure was based on the methodology used in previous studies that assessed the isodose-line overlap and volume of RILI after radiotherapy for stage I lung cancer [11]. Briefly, acute- and late-phase lung CT images were overlaid with the treatment planning using an intensity-based free-form DIR limited to the irradiation field using MIM Maestro (MIM Maestro ver. 6.1, MIM Software Inc., Cleveland, Ohio). To ensure DIR accuracy, five landmarks were placed along pulmonary vessels near the irradiation field – three on the affected (high-dose) side and two on the contralateral (low-dose) side – thereby sampling both high- and low-dose lung regions, and the target registration error at each landmark was confirmed to be ≤3 mm. In addition, visual quality assurance of the deformed dose overlay was performed for all cases. Initial RILI contours on follow-up CT were independently created in MIM by two board-certified radiation oncologists, one of whom (R.M.) had prior diagnostic radiology training and thoracic imaging expertise and was blinded to symptomatic outcome and the results of dose–volume analyses. During the consensus review process, discrepancies were resolved by directly modifying the contour set created by T.I. to generate the final consensus contour used for analysis. Because the pre-consensus independent contour sets were not preserved as separate analysable structures, formal inter-observer agreement metrics were not computed. The volume of RILI in both the acute- and late-phase CT scans was delineated using the contour drawing tool in MIM, and the corresponding volumes were measured. RILI on CT images was visually quantified on MIM using isodose-line overlap (%), measured in 10% increments from 10% to 100%. A lower isodose-line overlap value indicates that RILI extends into lower-dose regions and therefore reflects broader spatial spread of injury beyond the high-dose field; conversely, a higher value indicates that RILI is confined to higher-dose regions. The acute phase was analysed in all cases, while the late phase was assessed in 45 patients (78%). The thoracic CT images used for evaluation were obtained using a LightSpeed VCT 64-slice CT scanner (GE Healthcare, Waukesha, Wisconsin, USA) with a slice thickness of 5 mm.

For CT analysis, treatment-planning CT images acquired with a slice thickness of 2.5 mm were reconstructed with a 5-mm slice thickness to match the 5-mm follow-up CT images. To investigate the factors influencing the radiographic extent of RILI, analyses of the pretreatment lung condition and measurements of the lung dose (V5Gy–V60Gy) from dose-volume histograms (DVH) were conducted. Treatment planning thoracic CT images were analysed using imaging software (SYNAPSE VINCENT, Fujifilm, Tokyo, Japan) to assess lung volume, mean lung Hounsfield unit (HU) values, HU values of the lung lobe containing the primary lesion, and Goddard score. For all volumetric and HU measurements, the GTV was excluded from the regions of interest. The Goddard score is a visual, semi-quantitative method for classifying the severity of emphysema. Six images from both lungs, obtained from three slices at the level of the aortic arch, the carina, and 1 cm above the right diaphragm, were categorised with scores ranging between 0 and 4 as follows: a score of 0 indicated normal lung tissue, a score of 1 reflected LAA ≤25%, a score of 2 represented LAA between 25% and 50%, a score of 3 corresponded to LAA between 50% and 75%, and a score of 4 indicated LAA >75%. The total Goddard score was calculated by summing all six regions, yielding a possible range of 0–24 [12]. For analysis, patients were dichotomised into two groups: those with a Goddard score of 0 (no emphysema) and those with a score >0 (any emphysema). In addition, quantitative analysis of emphysema was performed by calculating the percentage of LAA throughout the entire lung volume using an attenuation threshold of −950 HU on CT. This quantitative LAA% served as a complementary measure to the Goddard score.

### Statistical analysis

The Shapiro–Wilk test was used to assess normality. Spearman’s rank correlation coefficients (ρ) were reported with two-sided *p*-values. Correlation strength was qualitatively interpreted as negligible (<0.10), weak (0.10–0.39), moderate (0.40–0.69), or strong (≥0.70). Continuous variables were compared between groups using the Wilcoxon rank-sum test. Independence between categorical variables was determined using the chi-squared test; Fisher’s exact test was used when expected counts were <5.

Receiver operating characteristic (ROC) curves were used to evaluate the ability of continuous variables to discriminate outcomes. Area under the curve (AUC) values were reported with 95% confidence intervals (CIs) using the DeLong method, and optimal cutoffs were identified *via* the Youden index.

Univariate and multivariable logistic regression analyses were performed to identify risk factors for symptomatic RILI. Because only 14 events were observed, the multivariable model was restricted to two predictors to limit overfitting. To reduce redundancy, ‘RILI extent’ was operationalised as RILI reaching the ≤25% isodose line (i.e. acute-phase RILI extending into lower-dose regions at or below the 25% isodose line, equivalent to ≤16.5 Gy for a 66-Gy prescription), and one dose–volume metric (V20Gy >20% or MLD >16 Gy) was chosen by Akaike information criterion (AIC). Odds ratios with 95% CIs and two-sided p-values were reported; complete-case analysis was used throughout. As a sensitivity analysis for the small number of events, Firth’s penalised-likelihood logistic regression was performed using the same covariates as the primary multivariable model, with the conventional logistic regression retained as the primary analysis. A two-sided *p*-value <0.05 was considered statistically significant. The R software (ver. 4.4.1, R Foundation for Statistical Computing, Vienna, Austria) was used for all statistical calculations.

## Results

Among the 58 patients, 47 were men and 11 were women, with a median age of 66 years (range, 43–80 years). The patient and tumour characteristics are summarised in Table 1. The median periods from the initiation of CCRT to acute and late CT were 6.2 months (range, 3–7.9 months) and 11.9 months (range, 9.5–14.3 months), respectively. Visual evaluation by isodose line of the 45 patients for whom late CT images were obtained showed a decrease in RILI isodose involvement in 26 patients compared to that in the acute phase, no change in 18, and an increase in one. The median RILI volume and isodose-line overlap were 159.5 mL and 40% (range: 10–90%) in the acute phase and 128 mL and 60% (range: 10–90%) in the late phase, respectively. The median reduction from acute phase to late phase in RILI volume was 27.7%. We analysed the association between the radiographic extent of RILI on CT and the site of the primary lesion (lower lobe or otherwise) in the acute and late phases and found a significant difference only in the volume of the acute phase (*p* = 0.01). Because the breath-hold technique was not employed during radiotherapy, tumours located in the lower lobes may have required larger irradiation fields due to respiratory motion, as reflected by a significantly higher V20Gy in lower-lobe tumours (median 30.45% vs 19.25%; *p* = 0.0059). Among the 14 patients with symptomatic RILI, 12 had grade 2 pneumonitis requiring oral corticosteroids and 2 had grade 3 pneumonitis requiring hospitalisation, supplemental oxygen, and corticosteroids; these corresponded to 20.7% and 3.4% of the total cohort, respectively. No grade ≥4 events occurred. In grade 2 or higher RILI (symptomatic RILI) cases, the median isodose-line overlap and volume in the acute phase were 20% and 273.9 mL, and in non-symptomatic cases, 40% and 136.2 mL, all statistically significant differences (isodose-line overlap: *p* < 0.001, volume: *p* < 0.001). In the late phase, the median value of the symptomatic RILI cases was 30% and 174.2 mL, and the median value of the non-symptomatic cases was 60% and 94.4 mL, both statistically significant differences (isodose-line overlap: *p* < 0.001, volume: *p* = 0.008). Baseline characteristics were compared between patients with and without symptomatic RILI, including age, pack-years, PTV volume, presence of emphysema defined by Goddard score (>0), %LAA (≥5%), and tumour location (lower lobe vs. others), with only pack-years showing a significant difference (*p* = 0.025) (Table 2).

**Table 1.** Patient and tumor characteristics.

Factors		Data
Sex		
	Male	47
	Female	11
Age (range)		Median 66 (43–80)
Location		
	Upper	39
	Middle	7
	Lower	12
Pathology		
	Adenocarcinoma	33
	Squamous cell carcinoma	18
	NSCLC	7
Smoking history		53
Pack-years (smokers only)		Median 45 (0.1–200)
Goddard		
	0–7	53
	8–15	5
	16–24	0
%LAA (−950 HU)		Median 0.4 (0–38)
T		
	1	13
	2	11
	3	13
	4	21
N		
	0	5
	1	5
	2	22
	3	26
Regimen		
	CDDP+VNR	30
	CDDP+TS-1	11
	CBDCA+PTX	9
	CBDCA	3
	CDDP+DTX	3
	CBDCA+PEM	1
	CDDP+ETP	1

**Table 2.** Comparison of baseline characteristics by symptomatic RILI.

Characteristic	No N = 44^1^	Yes N = 14^1^	*p*-value^2^
Age, years	66 (60–70)	67 (51–72)	0.8
Pack-years	46 (32–60)	34 (3–45)	0.025
PTV, mL	252 (175–370)	342 (212–452)	0.2
Goddard (0 vs >0)			0.5
0 (no emphysema)	29 (66%)	11 (79%)	
>0 (any emphysema)	15 (34%)	3 (21%)	
%LAA (−950 HU)			>0.9
<5%	35 (80%)	12 (86%)	
≥5%	9 (20%)	2 (14%)	
Tumor location			0.7
Other lobes	34 (77%)	12 (86%)	
Lower lobe	10 (23%)	2 (14%)	
^1^Median (Q1–Q3); n (%)			
^2^Wilcoxon rank sum test; Fisher’s exact test			

We analysed the correlation of RILI volume and isodose-line overlap with dosimetric parameters in both phases (Table 3; Supplementary Table S2). Correlation analyses demonstrated consistent dose–response relationships in both phases. In the acute phase, RILI volume showed strong positive correlations with lung dosimetric parameters across V10–V40 (*r* = 0.70–0.73, all *p* < 0.01), whereas the corresponding isodose-line overlap correlations were weaker (*r* = −0.44 to −0.49, *p* < 0.01). In the late phase, RILI volume exhibited moderate-to-strong positive correlations across V20–V50 (*r* = 0.69–0.76, all *p* < 0.01), while isodose-line overlap correlations remained moderate (*r* = −0.48 to −0.65, all *p* < 0.01). MLD also correlated significantly with RILI volume, though with slightly weaker coefficients (acute *r* = 0.66; late *r* = 0.61, both *p* < 0.01). Thus, correlation coefficients were consistently higher for volume than for isodose-line overlap throughout both phases.

**Table 3.** Correlation coefficients between the range of RILI and dosimetric parameters in the acute and late phases.

Phase	Measure	PTV volume	Lung -HU	pLung -HU	V5	V10	V15	V20	V25	V30	V35	V40	V45	V50	V55	V60	MLD
Acute	Isodose-line	r= −0.15 p = 0.25	r= −0.29 p = 0.03	r= −0.26 p = 0.05	r= −0.44 p < 0.01	r= −0.47 p < 0.01	r= −0.49 p < 0.01	r= −0.49 p < 0.01	r= −0.47 p < 0.01	r= −0.46 p < 0.01	r= −0.44 p < 0.01	r= −0.44 p < 0.01	r= −0.42 p < 0.01	r= −0.40 p < 0.01	r= −0.35 p < 0.01	r= −0.37 p < 0.01	r= −0.42 p < 0.01
	Volume	r = 0.33 *p* = 0.01	r = 0.16 *p* = 0.23	r = 0.43 *p* < 0.01	r = 0.67 *p* < 0.01	r = 0.70 *p* < 0.01	r = 0.71 *p* < 0.01	r = 0.71 *p* < 0.01	r = 0.72 *p* < 0.01	r = 0.73 *p* < 0.01	r = 0.73 *p* < 0.01	r = 0.71 *p* < 0.01	r = 0.68 *p* < 0.01	r = 0.65 *p* < 0.01	r = 0.56 *p* < 0.01	r = 0.57 *p* < 0.01	r = 0.66 *p* < 0.01
Late	Isodose-line	r= −0.14 *p* = 0.36	r= −0.27 *p* = 0.07	r= −0.19 *p* = 0.22	r= −0.61 *p* < 0.01	r= −0.62 *p* < 0.01	r= −0.62 *p* < 0.01	r= −0.65 *p* < 0.01	r= −0.63 *p* < 0.01	r= −0.61 *p* < 0.01	r= −0.58 *p* < 0.01	r= −0.56 *p* < 0.01	r= −0.50 *p* < 0.01	r= −0.48 *p* < 0.01	r= −0.41 *p* < 0.01	r= −0.39 *p* < 0.01	r= −0.55 *p* < 0.01
	volume	r = 0.39 *p* < 0.01	r = 0.25 *p* = 0.10	r = 0.39 *p* < 0.01	r = 0.60 *p* < 0.01	r = 0.66 *p* < 0.01	r = 0.67 *p* < 0.01	r = 0.69 *p* < 0.01	r = 0.70 *p* < 0.01	r = 0.72 *p* < 0.01	r = 0.74 *p* < 0.01	r = 0.76 *p* < 0.01	r = 0.75 *p* < 0.01	r = 0.72 *p* < 0.01	r = 0.61 *p* < 0.01	r = 0.65 *p* < 0.01	r = 0.61 *p* < 0.01

Abbreviations: PTV: Planning target volume; Vx: volume of normal lung receiving X Gy; MLD: mean lung dose.

Spearman’s rank correlation coefficients (ρ) and two-sided *p*-values are presented. Corresponding 95% confidence intervals and median ρ values are provided in Supplementary Table S2.

**Figure 3.**
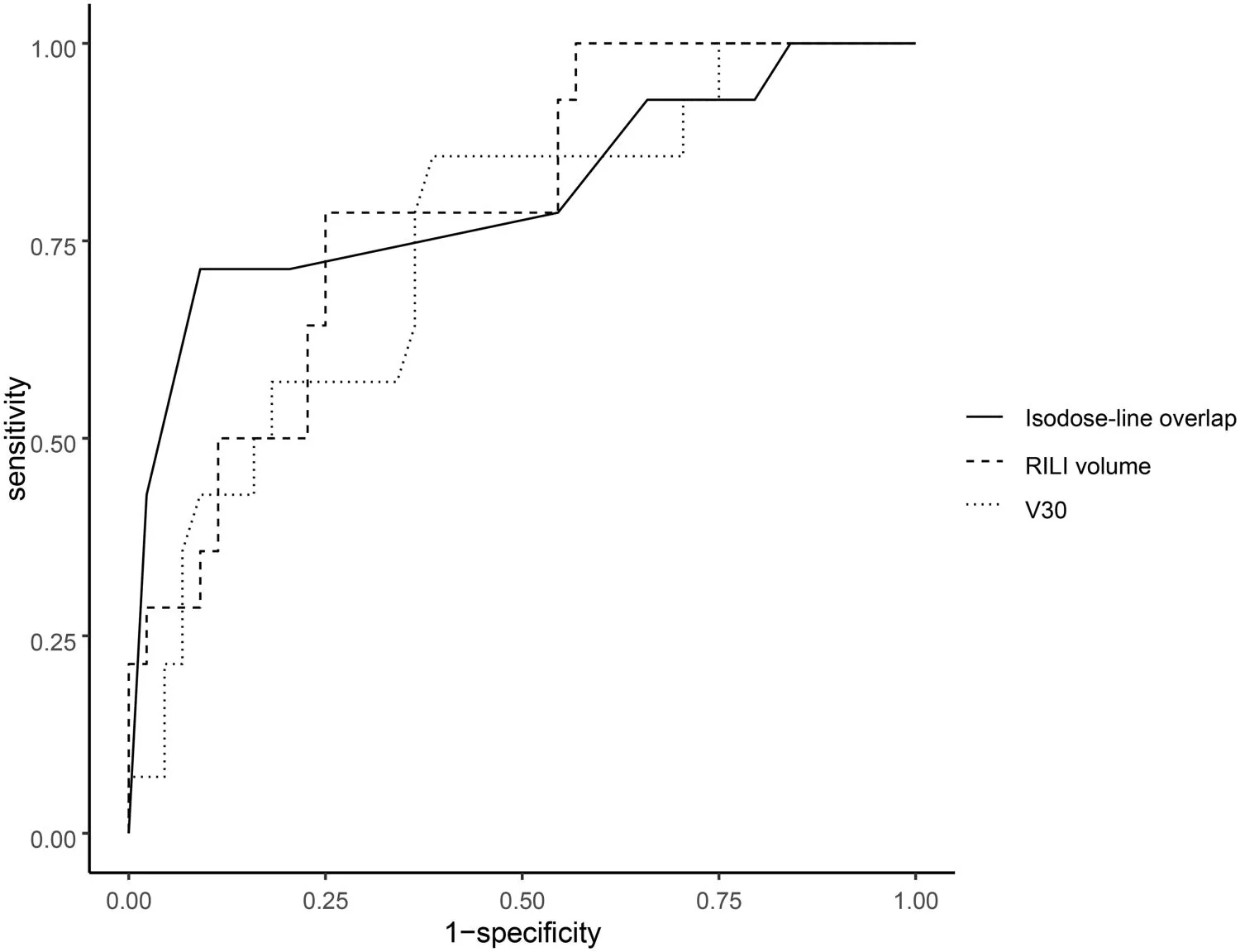
Receiver operating characteristic curves for isodose-line overlap (%), RILI volume, and V30Gy, with symptomatic RILI as the outcome in the acute phase. The AUCs were 0.81 (95% CI, 0.65–0.96) for acute-phase isodose-line overlap, 0.79 (95% CI, 0.66–0.92) for acute-phase RILI volume, and 0.75 (95% CI, 0.60–0.89) for V30Gy.

ROC analysis of lung dosimetric parameters (V5Gy–V60Gy and MLD) yielded a median AUC of 0.75; V30Gy performed best (cut-off 17.7%). For imaging-derived metrics, RILI volume (mL) and isodose-line overlap (%) in the acute phase, the AUC was 0.79 and 0.81, with optimal cut-off values of 220.9 mL and 25%, respectively. This indicates that patients whose acute-phase RILI reached the ≤25% isodose line of the treatment plan (≤16.5 Gy for a 66-Gy prescription) showed broader spatial spread of injury into lower-dose regions and were at higher risk of symptomatic RILI. Notably, symptomatic patients showed a significantly lower median isodose-line overlap value (20%) compared with asymptomatic patients (40%), confirming that a lower isodose-line overlap value reflects more extensive spatial spread of RILI into lower-dose regions. These three predictors are shown on a single ROC plot (Figure 3).

Parameters associated with symptomatic RILI were evaluated using univariate logistic regression (UVA). We selected a priori V20Gy (>35%, >25%, >20%), V5Gy (>65%), MLD (>20 Gy, >16 Gy, >10 Gy), age (>70 years), and sex (male) as factors based on previous studies and clinical relevance, and we added an isodose metric (RILI reaching the ≤25% isodose line), RILI volume (>221 mL) and pack-years based on this study. The threshold of V5Gy >65% was pre-specified based on established RILI risk metrics; because no patient exceeded this threshold in the present 3D-CRT cohort, V5Gy >40% was additionally explored post hoc based on evidence from IMRT-based cohorts suggesting that the effective threshold may be lower than the conventional constraint of 65%[13]. On UVA, V20Gy >20% (OR 7.89, 95% CI 1.58–39.55, *p* = 0.012), MLD >16 Gy (OR 5.19, 95% CI 1.43–18.79, *p* = 0.012), RILI reaching the ≤25% isodose line (OR 25.0, 95% CI 5.3–117.6, *p* < 0.001), and volume >221 mL (OR 11.0, 95% CI 2.59–46.78, *p* = 0.001) were significantly associated with symptomatic RILI. V5Gy >40% showed a non-significant trend toward association with symptomatic RILI (OR 2.86, 95% CI 0.83–9.81, *p* = 0.095). Because only 14 symptomatic events were available (EPV ≈ 7 for two covariates), we restricted the multivariable analysis (MVA) to two predictors. To reduce redundancy among dose–volume metrics and to represent RILI with a single variable, we retained the isodose metric and compared two candidate two-predictor MVA models – {V20Gy >20% + RILI reaching the ≤25% isodose line} and {MLD >16 Gy + RILI reaching the ≤25% isodose line}. The latter had a lower AIC and was selected as the primary MVA; in the final two-predictor MVA, only RILI reaching the ≤25% isodose line remained significant (OR 19.6, 95% CI 4.0–100, *p* = 0.0002). For clarity, UVA and MVA results are summarised side-by-side in Table 4. As a sensitivity analysis for the small number of events, Firth’s penalised-likelihood logistic regression was applied using the same two covariates as the primary multivariable model. Acute-phase RILI reaching the ≤25% isodose line remained associated with symptomatic RILI after adjustment for MLD >16 Gy (OR 15.4, 95% CI 3.7–74.8, *p* < 0.001), consistent with the conventional model (OR 19.6, 95% CI 4.0–100), although the wide confidence intervals support interpreting this finding as exploratory.

**Table 4.** Univariate and multivariable logistic regression analyses for symptomatic RILI.

Variable	UVA OR (95% CI)	UVA p	MVA OR (95% CI)	MVA p
V20Gy >20%	7.89 (1.58–39.55)	**0.012**	—	—
V20Gy >25%	2.58 (0.76–8.81)	0.131	—	—
V20Gy >35%	3.73 (0.66–21.09)	0.137	—	—
V5Gy >40%	2.86 (0.83–9.81)	0.095		
V5Gy >65%	NE†	NE	—	—
MLD >10 Gy	5.00 (1.00–25.02)	0.050	—	—
MLD >16 Gy	5.19 (1.43–18.79)	**0.012**	2.7 (0.57–12.44)	0.20
MLD >20 Gy	3.50 (0.45–27.53)	0.234	—	—
Age >70	1.36 (0.35–5.28)	0.657	—	—
Sex (male)	0.284 (0.07–1.14)	0.076	—	—
Pack-years	0.988 (0.97–1.01)	0.275	—	—
RILI reaching the ≤25% isodose line	25.0 (5.3–117.6)	**<0.001**	19.6 (4.0–100)	**0.0002**
Volume >221 mL	11.0 (2.59–46.78)	**0.001**	—	—
Abbreviations: UVA: univariate logistic regression; MVA: multivariable logistic regression; OR: odds ratio; CI: confidence interval; NE: not estimable. †Not estimable because no patients exceeded the predefined threshold of V5Gy >65%				

## Discussion

In this study, we used DIR to quantify RILI volume and isodose-line overlap in the acute and late phases and to examine their association with dosimetric parameters. Our results indicated a significant correlation between these RILI metrics and pulmonary dosimetric parameters, suggesting a dose-dependent relationship. In addition, the study revealed the following new findings: 1) RILI volume decreased by approximately 28% from the acute to late stage; 2) symptomatic RILI showed significantly greater RILI volume and broader spatial spread into lower-dose regions than non-symptomatic RILI; and 3) on MVA, RILI reaching the ≤25% isodose line was an exploratory imaging marker for symptomatic RILI in this cohort.

This study demonstrated a 28% reduction in RILI volume from the acute to the late phase. Pulmonary fibrosis, as observed in late-phase RILI, follows RP and manifests on imaging as diminished volume, linear scarring, consolidation, and traction bronchiectasis [14,15]. Radiological changes generally stabilise within 1–2 years post-irradiation, and CT-based lung density changes in patients with stage III NSCLC treated with CCRT have been shown to evolve in a time- and dose-dependent manner during follow-up [16]. On the other hand, Ma et al. described that the increase in lung density on CT after RT progresses over the first 6 months of treatment, reaching a plateau at approximately 6–12 months [7]. The differences between their study and ours are that 1) not all patients had lung cancer (81%), 2) 49% received chemotherapy, and 3) no registration technique was used for alignment, which may have caused the variation in results.

Second, this study revealed a correlation between symptoms and RILI images. The RILI volume in symptomatic patients during the acute phase was approximately twice that of asymptomatic patients. Previous studies using conventional fractionated radiation therapy with 3D-CRT reported that when the radiation dose to the chest is less than 20 Gy, CT imaging in the acute phase shows only a few findings suggestive of RILI, often within an isodose line of 27 Gy or more [7,17]. These results support our results, with a median isodose-line overlap of 20% (=13.2 Gy) in patients with symptomatic RILI and 40% (=26.4 Gy) in non-symptomatic RILI. Isodose-line overlap was recorded in 10% increments, and ROC analysis identified an optimal cutoff lying between the 20% and 30% categories, expressed as RILI reaching the ≤25% isodose-line threshold. The ≤25% isodose level corresponds to approximately 16.5 Gy for a 66-Gy prescription, which is slightly below the dose range previously reported as the lower boundary for radiographic RILI; extension of RILI into this low-dose region may therefore indicate unusually broad spatial spread of lung injury rather than conventional high-dose–confined radiation change. Injury extending beyond the high-dose region may reflect propagation of cytokine-mediated inflammation into low-dose lung, regional differences in parenchymal susceptibility, and inter-individual radiosensitivity, rather than dose deposition alone. Acute RILI following radiotherapy with intensity-modulated radiation therapy (IMRT) for stage I NSCLC has been reported to occur within 60 Gy, which differs from the results of our study [11]. This discrepancy likely reflects the higher dose conformity of IMRT and the smaller planning target volumes (PTV) in early-stage disease. The relationship between the radiographic changes in RILI and its symptoms remains controversial.

All pulmonary dosimetric parameters were significant predictors of RILI volume and isodose-line overlap in this study. A Japanese report predicting symptomatic RILI after CCRT for NSCLC reported that age >68 years was a risk factor [4]. The median age in our study was 66 years, and the definition of symptomatic RILI differed from that of grade 3 or higher, which may have influenced the analysis. Numerous reports have linked increased tumour volume to a higher incidence of RILI; however, these results should be interpreted with caution [18]. In stage 1 NSCLC, an increase in tumour volume directly leads to an increase in the lung dose. In this study, however, stage III NSCLC was targeted, with most cases (N2/3, 83%) exhibiting lymph node enlargement in the mediastinum or supraclavicular fossa. Consequently, the PTV volume does not solely correspond to pulmonary lesions, which may account for the lack of a significant difference in the PTV volume observed here. As reported in the Results (Table 2), emphysema (Goddard score >0 or %LAA ≥5%) was not a risk factor for symptomatic RILI in this cohort. This finding is consistent with the view that emphysematous lungs may have reduced functional parenchyma susceptible to radiation injury. Nevertheless, the role of emphysema in RILI after CCRT for locally advanced NSCLC remains controversial [4,19]. A Chinese study reported an increased risk of symptomatic RILI when radical radiotherapy was administered to patients with NSCLC and severe emphysema [19]. A possible difference between their study and ours is the method used to evaluate emphysema. In our study, emphysematous changes in the whole lung were evaluated using an image analysis software, whereas in the Chinese study, the evaluation was based on a single axial cross-section. In addition, the small number of patients with moderate emphysema in our study (five patients) may have influenced the analysis.

This study has several limitations. First, this study focused on RILI symptoms and changes on CT imaging; respiratory function test results or SPECT images were not obtained in all cases. Respiratory function tests were performed in all patients before radiotherapy but not during the follow-up period; therefore, they could not be contrasted with the images. This lack of longitudinal functional data limited our ability to fully correlate radiological changes with clinical respiratory outcomes, a limitation inherent to the retrospective design. Although abdominal compression was used for motion management, free-breathing acquisition may leave residual respiratory-motion–related registration uncertainty, particularly in the lower lobes; this is acknowledged as a limitation. Although initial independent contouring was performed by two readers, the pre-consensus contour sets were not preserved as separate analysable structures after consensus editing in MIM; therefore, formal inter-observer agreement metrics could not be quantified. Residual inter-observer variability in RILI contour delineation remains a limitation. Overall, the small number of symptomatic events (*n* = 14), the single-institution design, residual inter-observer variability, and the historical 3D-CRT, pre-durvalumab treatment era together limit the statistical robustness and generalisability of these findings, which should be regarded as hypothesis-generating. In particular, the wide confidence intervals around the logistic-regression estimates indicate limited precision; therefore, the ≤25% isodose-line finding should be considered hypothesis-generating rather than a robust predictive threshold. We are initiating a prospective study to address this gap by combining pre- and post-treatment respiratory function tests with four-dimensional CT (JSPS KAKENHI Grant Numbers JP21K15812 and JP26K19006). Second, 3D-CRT was used as the irradiation method in all cases, and IMRT was not used. Although 3D-CRT is no longer the standard technique for locally advanced NSCLC, its use in this study provided a relatively homogeneous dose distribution pattern, allowing clearer assessment of spatial concordance between planned dose and CT-defined injury. Previous secondary analysis of NRG Oncology RTOG 0617 demonstrated that IMRT was associated with lower rates of severe pneumonitis than 3D-CRT in locally advanced NSCLC [20]. This cohort should therefore be interpreted as a controlled reference dataset for radiation-induced lung injury, rather than a direct predictor of toxicity in contemporary IMRT-based practice. Whether the dosimetric-radiographic correlations observed here translate to IMRT-treated patients warrants prospective validation, given that IMRT reduces medium-dose exposure (V20–40 Gy) while increasing low-dose exposure (V5Gy), and the radiographic distribution of RILI may differ accordingly. In the present 3D-CRT cohort, V5Gy >65% was not estimable as all patients remained below this threshold; however, a lower threshold of V5Gy >40% showed a non-significant trend toward association with symptomatic RILI (OR 2.86, *p* = 0.095), consistent with reports in IMRT-based cohorts suggesting that the conventional V5 constraint of <65% may be excessively high for symptomatic radiation pneumonitis [13]. Third, although treatment-planning CT images were acquired with a slice thickness of 2.5 mm, they were reconstructed with a 5-mm slice thickness for CT analysis to match the 5-mm follow-up CT images. This thickness can limit detection of subtle morphologic changes such as ground-glass opacity, reticulation, or early traction bronchiectasis, and may bias quantitative emphysema estimates derived from low-attenuation thresholds because slice thickness and reconstruction kernel influence lung densitometry. Our objective in this study was to explore how the radiological extent of RILI on CT and the presence of symptoms relate to dose distribution. The impact of 5-mm slices on the analysis is considered to be smaller than in the case of microstructural morphologic changes.

In this study, we analysed the radiographic extent of RILI on CT in patients with stage III NSCLC under observation without further treatment following definitive CCRT. We acknowledge that this cohort does not reflect the current clinical standard, in which durvalumab consolidation after CCRT is routinely administered per the PACIFIC trial. The exclusion of ICI-treated patients was intentional: concurrent ICI-associated pneumonitis would have precluded unambiguous dosimetric-radiographic attribution of CT changes to radiation alone. The dosimetric thresholds and morphologic patterns described here therefore represent a necessary reference baseline for pure radiation-related injury. Future prospective studies should compare RILI morphology and isodose-line overlap between patients receiving CCRT alone versus CCRT followed by durvalumab, using the DIR methodology described here, to disentangle radiation- and ICI-related pulmonary toxicity in the era of combined modality immunotherapy. Future work should pursue prospective multicentre validation, integration with longitudinal pulmonary function testing, radiomics and machine-learning–based prediction, and direct comparison of radiation- versus immunotherapy-related lung injury using the present DIR framework. External validation in larger, multicentre cohorts is required before any clinical implementation of the proposed marker.

## Conclusions

Dose-registered CT analysis demonstrated consistent spatial associations between planned lung dose and CT-defined radiation-induced lung injury in a uniform pre-durvalumab 3D-CRT cohort. RILI volume decreased by approximately 28% from the acute to the late phase. Acute-phase RILI reaching the ≤25% isodose line (≤16.5 Gy for a 66-Gy prescription) was an exploratory imaging marker for symptomatic RILI in this cohort (AUC 0.81) that remained significant on multivariable analysis. These findings should be interpreted as hypothesis-generating and as a dosimetric-radiographic reference for future studies incorporating modern radiotherapy techniques and consolidation immunotherapy.

## Supplementary Material

Supplementary_Table_S2_45cases.docx

Supplementary_Table_S1_R2.docx

## Data Availability

The datasets generated and/or analysed during the current study are not publicly available because they contain potentially identifiable clinical and imaging data, but are available from the corresponding author on reasonable request and with institutional approval.
