# Supplementary material for: Dose-Registered CT assessment of radiation-induced lung injury after chemoradiotherapy for stage III NSCLC
Source: Ann Med. 2026 Sep 10;58(1):2721850. doi: 10.1080/07853890.2026.2721850 (PMC13573539; doi:10.1080/07853890.2026.2721850)
Supplement: Supplementary file 1 [file IANN_A_2721850_SM1605.docx]

**Supplementary Table S1. CT-based definitions and examples of radiation-induced lung injury (RILI)**

Terminology and patterning follow Koenig et al.

| Phase | CT pattern | Morphologic definition (CT) | Distribution & dose–geometry | Timing |
| --- | --- | --- | --- | --- |
| Acute | Ground-glass opacity (GGO) | Increased lung attenuation with preserved bronchovascular markings; typical imaging correlate of acute radiation pneumonitis. | Confined to ~2‑cm peritumoral high‑dose band; borders mirror isodose (straight/geographic). | Within ~3 months post‑RT completion |
| Acute–Late | bandlike consolidation | Vessel‑obscuring consolidation; accompanies/replaces GGO acutely, may persist within fibrosis. | Conforms to field or abuts edge; geometry reflects beam arrangement and dose fall‑off. | Acute ≤6 months; may persist at approximately 12 months as a fibrotic component. |
| Acute | Nodule-like pneumonitis | Small nodular lung opacities; acute radiation pneumonitis phenotype. | In-field, peritumoral; conforms to high-dose region. | Within ~3 months; typically resolves by ~6 months. |
| Late | Modified conventional fibrosis | Consolidation with parenchymal volume loss and traction bronchiectasis; less extensive than wide‑field RT fibrosis. | Confined to the treated volume; **less extensive than conventional fibrosis**; mirrors multi-beam dose gradient. | Established/stable by ~12 months. |
| Late | Masslike fibrosis | Focal masslike consolidation with traction bronchiectasis at the original tumor site; can mimic recurrence. | Matches maximal isodose shell around tumor; often within ~2‑cm peritumoral band. | At approximately 12 months; typically stable or decreases. |
| Late | Scarlike fibrosis | Linear band (≤10 mm thick) with moderate–marked parenchymal volume loss. | Linear streak at original site tracking beam geometry/dose fall‑off. | At approximately 12 months; long-term stability typical. |
| Late | Architectural distortion / reticulation | Distortion with reticulation, pleural retraction, traction bronchiectasis. | Follows irradiated geometry and dose gradient (field‑conforming borders). | Established at approximately 12 months. |

Notes: Phase is labeled as “Acute,” “Late,” or “Acute–Late” when a finding spans both phases. This table aligns with the manuscript text: acute RILI was assessed on CT obtained approximately 6 months after RT initiation, and late RILI was assessed on CT obtained approximately 12 months after RT initiation.
