# Supplementary material for: Dose-Registered CT assessment of radiation-induced lung injury after chemoradiotherapy for stage III NSCLC
Source: Ann Med. 2026 Sep 10;58(1):2721850. doi: 10.1080/07853890.2026.2721850 (PMC13573539; doi:10.1080/07853890.2026.2721850)
Supplement: Supplementary file 2 [file IANN_A_2721850_SM1606.docx]

**Supplementary Table S2. Spearman correlation coefficients with 95% confidence intervals**

Spearman’s rank correlation coefficients (ρ), Fisher z-transformed 95% confidence intervals, and two-sided p-values are shown. Acute-phase analyses included 58 patients. Late-phase analyses included the same 45 patients with evaluable late-phase isodose-line overlap and RILI volume. Median ρ values were calculated across V5Gy–V60Gy and mean lung dose.

**A. Acute phase (n = 58)**

| **Variable** | **Isodose-line overlap, ρ (95% CI)** | **p-value** | **RILI volume, ρ (95% CI)** | **p-value** |
| --- | --- | --- | --- | --- |
| PTV volume | −0.15 (−0.40 to 0.11) | 0.246 | 0.33 (0.08 to 0.54) | 0.011 |
| Lung HU | −0.29 (−0.51 to −0.04) | 0.027 | 0.16 (−0.10 to 0.40) | 0.231 |
| pLung HU | −0.26 (−0.49 to 0.00) | 0.053 | 0.43 (0.19 to 0.62) | <0.001 |
| V5Gy | −0.44 (−0.63 to −0.21) | <0.001 | 0.67 (0.50 to 0.79) | <0.001 |
| V10Gy | −0.47 (−0.65 to −0.25) | <0.001 | 0.70 (0.55 to 0.81) | <0.001 |
| V15Gy | −0.49 (−0.67 to −0.27) | <0.001 | 0.71 (0.56 to 0.82) | <0.001 |
| V20Gy | −0.49 (−0.66 to −0.26) | <0.001 | 0.71 (0.56 to 0.82) | <0.001 |
| V25Gy | −0.47 (−0.65 to −0.24) | <0.001 | 0.72 (0.56 to 0.82) | <0.001 |
| V30Gy | −0.46 (−0.64 to −0.23) | <0.001 | 0.73 (0.58 to 0.83) | <0.001 |
| V35Gy | −0.44 (−0.62 to −0.20) | <0.001 | 0.73 (0.58 to 0.83) | <0.001 |
| V40Gy | −0.44 (−0.63 to −0.21) | <0.001 | 0.71 (0.56 to 0.82) | <0.001 |
| V45Gy | −0.42 (−0.62 to −0.19) | <0.001 | 0.68 (0.52 to 0.80) | <0.001 |
| V50Gy | −0.40 (−0.60 to −0.16) | 0.002 | 0.65 (0.47 to 0.78) | <0.001 |
| V55Gy | −0.35 (−0.56 to −0.11) | 0.006 | 0.56 (0.36 to 0.72) | <0.001 |
| V60Gy | −0.37 (−0.57 to −0.12) | 0.005 | 0.57 (0.37 to 0.72) | <0.001 |
| MLD | −0.42 (−0.61 to −0.18) | <0.001 | 0.66 (0.48 to 0.78) | <0.001 |

**B. Late phase (n = 45)**

| **Variable** | **Isodose-line overlap, ρ (95% CI)** | **p-value** | **RILI volume, ρ (95% CI)** | **p-value** |
| --- | --- | --- | --- | --- |
| PTV volume | −0.14 (−0.42 to 0.16) | 0.355 | 0.39 (0.11 to 0.62) | 0.007 |
| Lung HU | −0.27 (−0.53 to 0.02) | 0.069 | 0.25 (−0.05 to 0.50) | 0.100 |
| pLung HU | −0.19 (−0.46 to 0.12) | 0.221 | 0.39 (0.10 to 0.62) | 0.009 |
| V5Gy | −0.61 (−0.77 to −0.39) | <0.001 | 0.60 (0.37 to 0.76) | <0.001 |
| V10Gy | −0.62 (−0.78 to −0.40) | <0.001 | 0.66 (0.45 to 0.80) | <0.001 |
| V15Gy | −0.62 (−0.77 to −0.40) | <0.001 | 0.67 (0.47 to 0.81) | <0.001 |
| V20Gy | −0.65 (−0.79 to −0.43) | <0.001 | 0.69 (0.50 to 0.82) | <0.001 |
| V25Gy | −0.63 (−0.78 to −0.41) | <0.001 | 0.70 (0.51 to 0.82) | <0.001 |
| V30Gy | −0.61 (−0.76 to −0.38) | <0.001 | 0.72 (0.54 to 0.84) | <0.001 |
| V35Gy | −0.58 (−0.75 to −0.34) | <0.001 | 0.74 (0.58 to 0.85) | <0.001 |
| V40Gy | −0.56 (−0.73 to −0.32) | <0.001 | 0.76 (0.60 to 0.86) | <0.001 |
| V45Gy | −0.50 (−0.69 to −0.24) | <0.001 | 0.75 (0.59 to 0.86) | <0.001 |
| V50Gy | −0.48 (−0.68 to −0.21) | <0.001 | 0.72 (0.54 to 0.84) | <0.001 |
| V55Gy | −0.41 (−0.62 to −0.13) | 0.006 | 0.61 (0.39 to 0.77) | <0.001 |
| V60Gy | −0.39 (−0.61 to −0.11) | 0.008 | 0.65 (0.45 to 0.80) | <0.001 |
| MLD | −0.55 (−0.73 to −0.31) | <0.001 | 0.61 (0.39 to 0.77) | <0.001 |

**C. Median ρ across V5Gy–V60Gy and MLD**

| **Outcome** | **Median ρ** | **Range of ρ** |
| --- | --- | --- |
| Acute isodose-line overlap | −0.44 | −0.49 to −0.35 |
| Acute RILI volume | 0.70 | 0.56 to 0.73 |
| Late isodose-line overlap | −0.58 | −0.65 to −0.39 |
| Late RILI volume | 0.69 | 0.60 to 0.76 |

Abbreviations: CI, confidence interval; HU, Hounsfield unit; MLD, mean lung dose; PTV, planning target volume; RILI, radiation-induced lung injury; VxGy, percentage of normal lung volume receiving at least x Gy.
